# Cauliflower Ear in Lepromatous Leprosy

**DOI:** 10.4269/ajtmh.21-0661

**Published:** 2021-09-27

**Authors:** Vijayasankar Palaniappan, Karthikeyan Kaliaperumal

**Affiliations:** Department of Dermatology, Venereology and Leprosy, Sri Manakula Vinayagar Medical College and Hospital, Pondicherry, India

**Figure 1.**
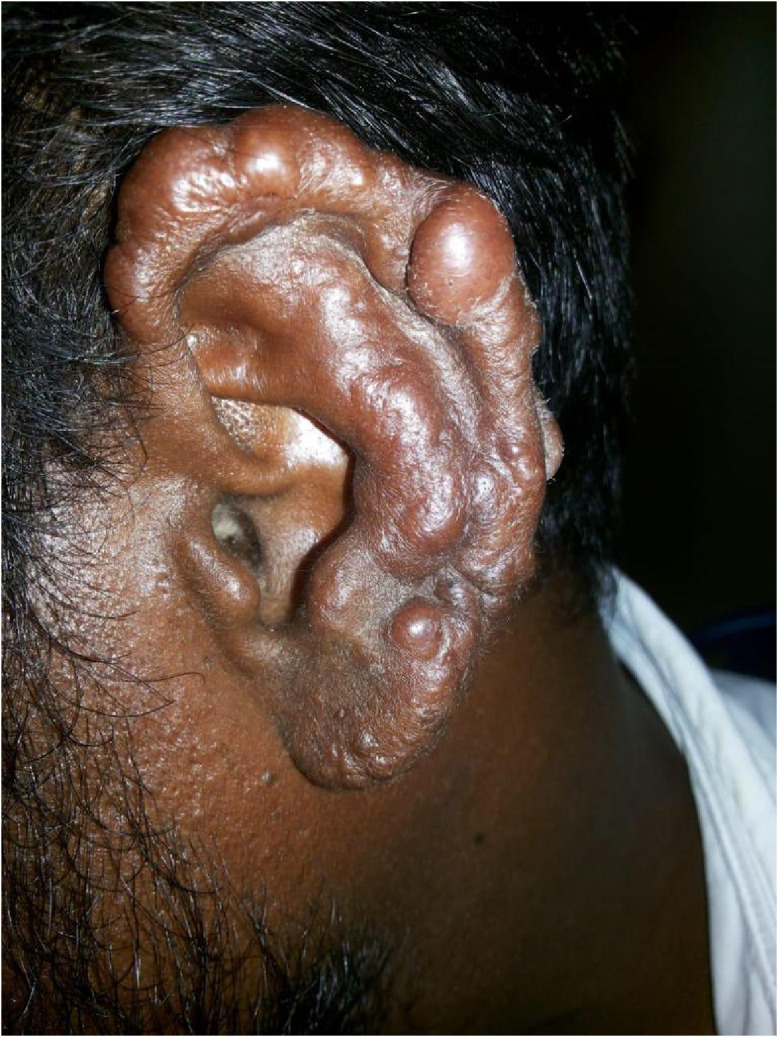
Multiple, well-defined, skin-colored papules and nodules over external ear. This figure appears in color at www.ajtmh.org.

A 49-year-old man presented with asymptomatic, raised lesions over his left ear for three years. On physical examination, he had diffuse infiltration and thickening with multiple well-defined, skin-colored, firm, nontender papules and nodules of size ranging from 0.3 cm to 1.3 cm over the helix, antihelix, lobule, and tragus of left ear, simulating a cauliflower appearance (Figure 1). There was no regional lymphadenopathy. He also had generalized xerosis with multiple, ill-defined, hypochromic macules over his trunk, arms, and thighs. Peripheral nerve examination showed thickening of left ulnar nerve with hypoanesthesia along its distribution. No motor deficits were found in the clinical evaluation.

**Figure 2.**
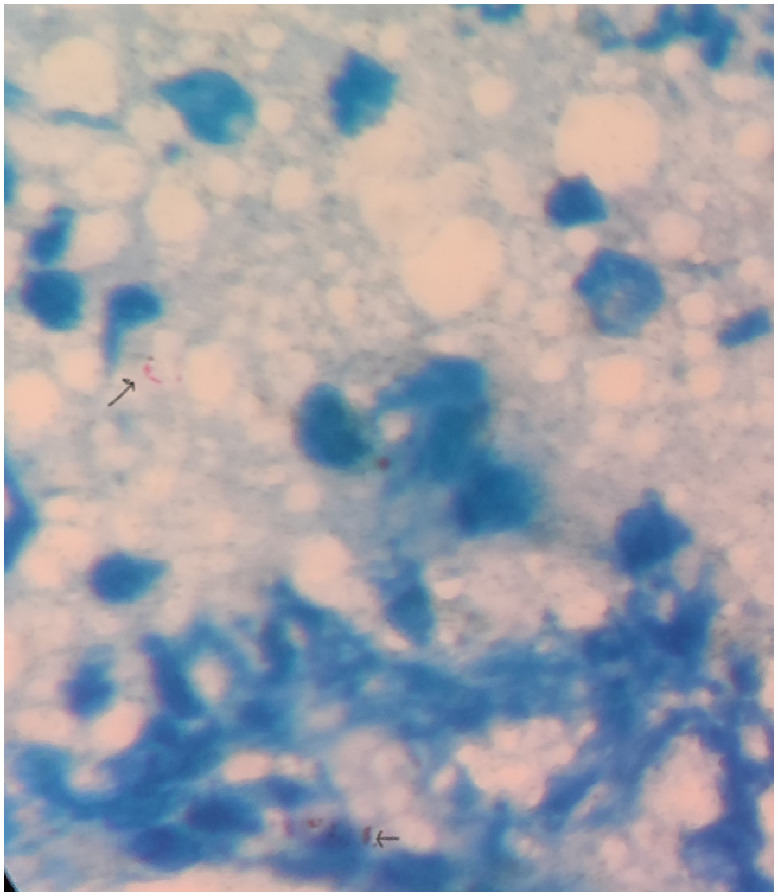
Slit skin smear—The arrows show acid-fast bacillus. This figure appears in color at www.ajtmh.org.

**Figure 3.**
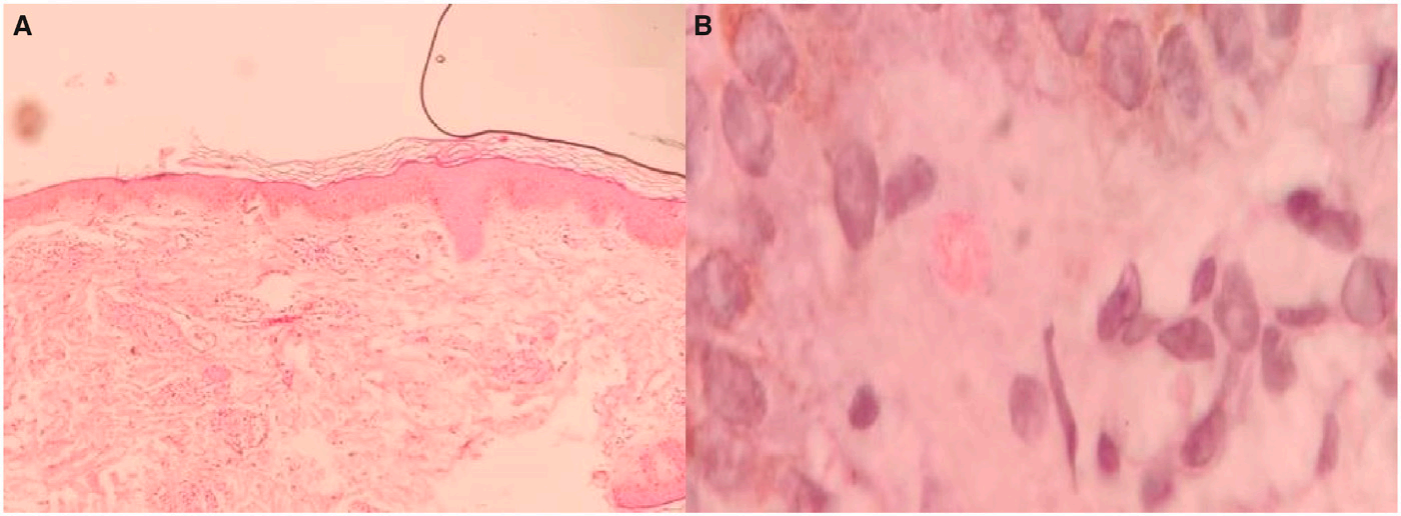
(A) Hematoxylin and eosin (H&E) stain—Low-power microscopic view shows epidermal atrophy with clear grenz zone. (**B**) H&E stain—High-power microscopic view shows lymphocytes and histiocytes in the dermis. This figure appears in color at www.ajtmh.org.

**Figure 4.**
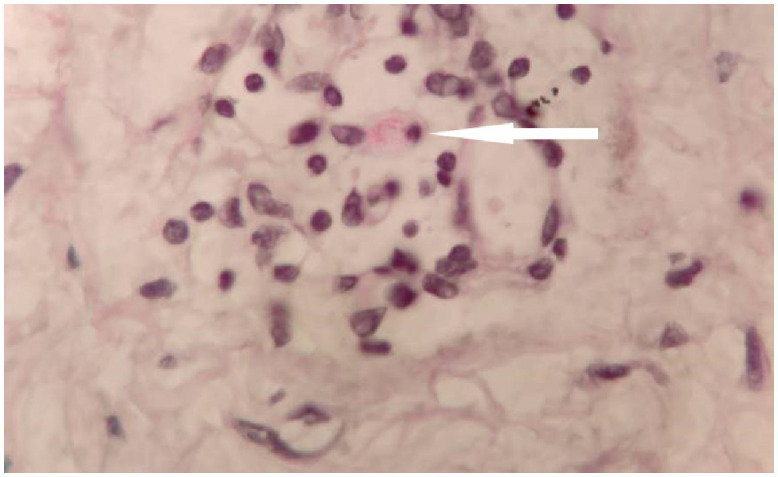
Fite-Faraco stain showing collection of *Mycobacterium leprae* bacillus (arrow), with a bacillary index of 5 + (×100 magnification). This figure appears in color at www.ajtmh.org.

Slit skin smear tests of pinna were performed. It revealed acid-fast bacilli (AFB) of *Mycobacterium leprae* with a bacteriological index of 3+ (Figure 2). Incisional biopsy from a nodule revealed macrophages, and lymphocytes diffusely distributed in the dermis with a free Grenz zone (Figure 3). Fite-Faraco stain (Special stain for *M. leprae*) was positive; with a bacillary index of 5+ (Figure 4). The clinical and laboratorial findings were consistent with features of lepromatous leprosy. The patient was started on WHO—Multi Drug Therapy regimen containing rifampicin, clofazimine, and dapsone.

*Mycobacterium leprae* has the predilection to involve cooler body sites such as pinna and lobule.^1^ Infiltration, nodule formation, ulceration with a “nibbled” or “rat-bitten” defect, megalobule, and auricular chondritis are the reported external ear manifestations of leprosy.^2^ Cases of leprosy with isolated pinna involvement also have been reported.

“Cauliflower ear” refers to a peculiar appearance of the external ear secondary to inflammation/infection.^3^ The differential diagnosis of cauliflower ear includes trauma/hematoma, lupus pernio (sarcoidosis), perniosis, lupus vulgaris, multicentric reticulohistiocytosis, lymphyoctyoma cutis, primary lymphoma, cutaneous leishmaniasis, Hansen’s disease, Rosai-Dorfman disease, relapsing polychondritis, and auricular pseudocyst.^4^

Leprosy should be considered as a differential diagnosis for any infiltrated, nodular lesions of external ear. Timely diagnosis and treatment initiation can prevent this kind of disfigurement that is responsible for stigma associated with leprosy.
